# Dysuria due to benign prostatic hyperplasia of the median lobe with ketamine-associated uropathy in a young male

**DOI:** 10.1186/s12894-019-0524-y

**Published:** 2019-10-30

**Authors:** Zhangcheng Liao, Zhao Wang, Zhongyuan Jin, Zhengyan Tang

**Affiliations:** 1 Department of Urology, Xiangya Hospital, Central South University, Changsha, Hunan China; 2 Provincial Laboratory for Diagnosis and Treatment of Genitourinary System Disease, Changsha, China; 3 Department of Pathology, Xiangya Hospital, Central South University, Changsha, Hunan China; 4 National Clinical Research Center for Geriatric Disorders, Changsha, Hunan China

**Keywords:** Dysuria, Young male, Benign prostatic median lobe hyperplasia, Ketamine-associated uropathy

## Abstract

**Background:**

Benign prostatic hyperplasia (BPH) rarely occurs in children or young males. In this case report, a 29-year-old male patient diagnosed with BPH coexisting with ketamine-associated uropathy was reported to investigate the possible relationship between BPH and ketamine-associated uropathy as well as therapeutic strategies.

**Case presentation:**

A 29-year-old male patient with a 3-year history of ketamine inhalation, complaining of dysuria with frequency and urgency, was admitted. Hydronephrosis, hydroureters, uneven bladder wall thickening and a tumour located in the outlet of the bladder were detected with computed tomography (CT). The patient agreed to cystoscopy under general anaesthesia. A spherical tumour with a diameter of approximately 2 cm was found to originate from the median lobe of the prostate and follicular lesions were diffusely distributed on the right bladder wall. The tumour and follicular lesions in the bladder were resected successfully, and pathology demonstrated BPH and chronic inflammation of the mucous membranes separately. The patient quit ketamine completely during the one-year follow-up. Dysuria was relieved completely and no tumour or follicular neoplasm recurrence was found.

**Contribution:**

Inflammation in the urothelium, as a direct or indirect consequence of ketamine, may contribute to the development of BPH. Both surgical interventions to remove obstruction and ketamine cessation are necessary approaches.

## Background

Benign prostatic hyperplasia (BPH) is a common disease that usually develops in middle-aged and older men. The occurrence of BPH in children or young men seems to be very rare. This report presents a case of BPH with ketamine-associated uropathy in a young patient which, to our knowledge, has never been reported before.

## Case presentation

**Fig. 1:**
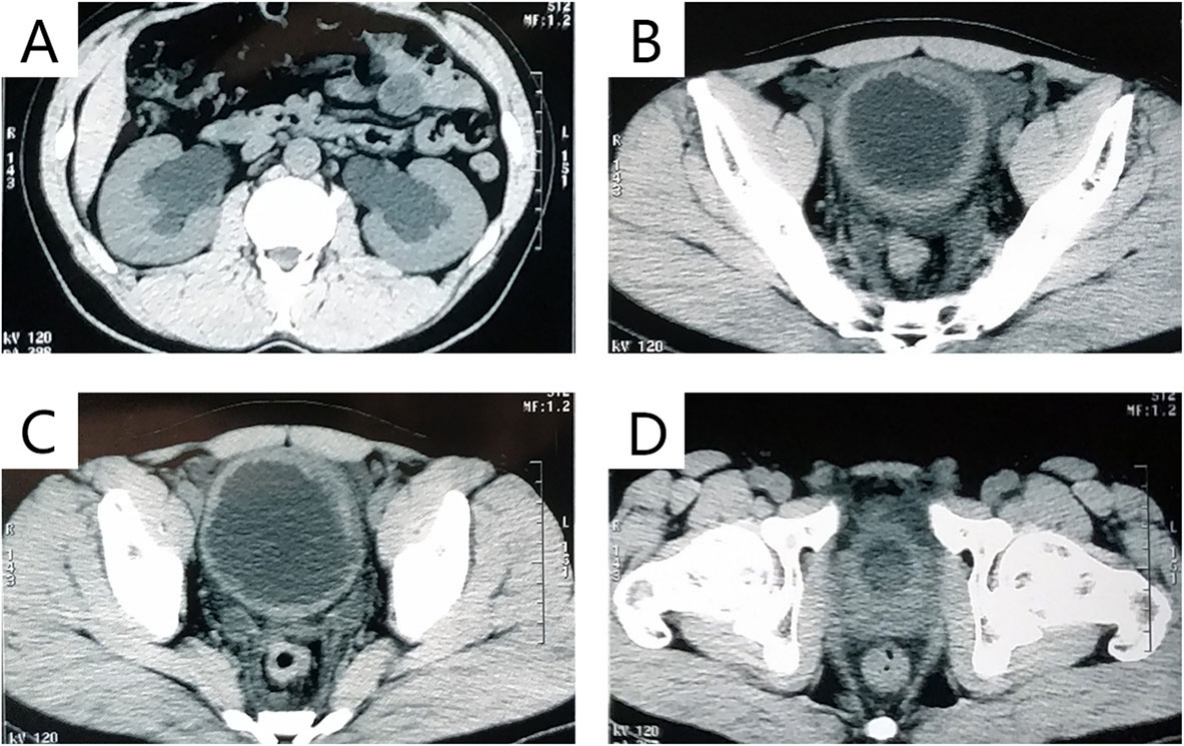
**a** Bilateral hydronephrosis. **b** and **c** Uneven bladder wall thickening with bladder diverticulum. **d** A round vague shadow near the bladder outlet

**Fig. 2:**
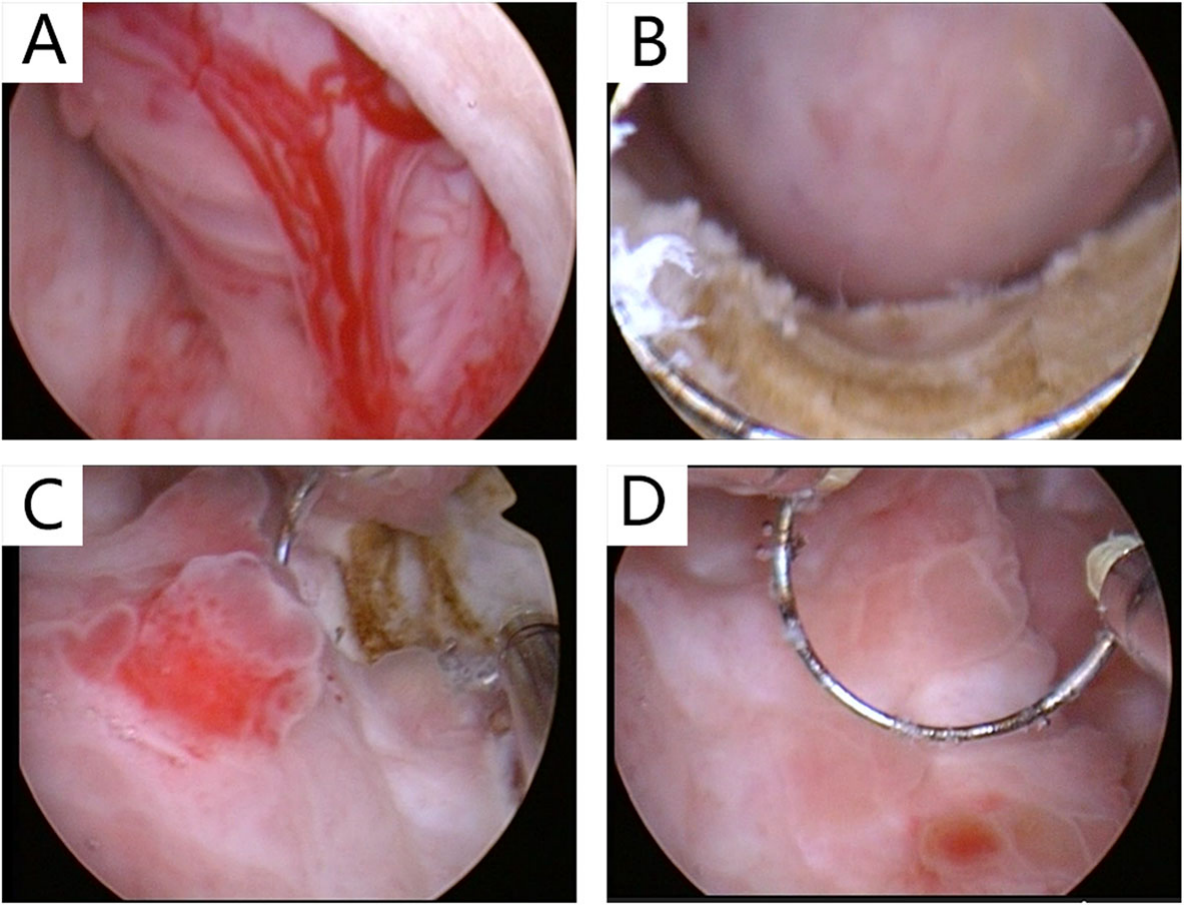
**a** The enlarged median lobe of the prostate protruding into the urethra and cyst. **b** The enlarged median lobe of the prostate and the surgical wound after TURP. **c** and **d** A fair amount of follicular neoplasm located on the right wall of the bladder

**Fig. 3:**
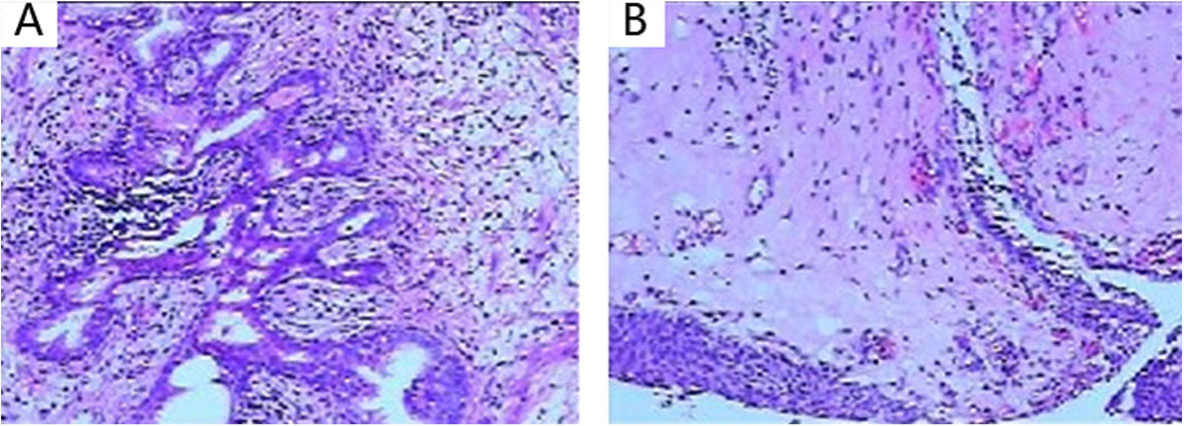
**a** Pathology of prostate tissues showing benign prostatic hyperplasia involving both the gland and stroma. The hyperplasic glands are well-differentiated with eosinophilic crystals in the lumen. **b** Pathology of follicular lesions showing chronic inflammation with denudation or loss of epithelium and marked inflammatory infiltration

A 29-year-old male patient was admitted on April 2017 because of dysuria with frequency and urgency. The patient began quitting ketamine for one year after 3 years of ketamine inhalation (once weekly at first and then once every 2–3 days up to a maximum of approximately 10 g/day). Duplex ultrasonography of the urinary system showed bilateral hydronephrosis and hydroureters, a thick-walled bladder and a post-void residual volume of approximately 100 ml. Computed tomography (CT) showed moderate bilateral hydronephrosis and hydroureters (Fig. 1a) as well as uneven bladder wall thickening with bladder diverticulum (Fig. 1b and c). A round, vague shadow approximately 2 cm in diameter near the bladder outlet (Fig. 1d) was detected after the CT image had been analysed carefully, but it was hard to distinguish its origin. The patient agreed to cystoscopy under general anaesthesia. A spherical tumour with a diameter of approximately 2 cm was found to originate from the median lobe of the prostate, obstructing the prostatic urethra (Fig. 2a), which impeded further progress with cystoscopy. The enlarged median lobe of the prostate was removed with transurethral resection of the prostate (TURP) completely and successfully (Fig. 2b). The bladder capacity was measured to be 200 ml (average of three measurements). Trabeculation and multiple bladder diverticula were visible on the rough mucosal surface of the bladder. On the right wall of the bladder, there was a significant amount of follicular neoplasm (Fig. 2c and d). Follicular lesions were resected completely. The pathology of the enlarged median lobe of the prostate demonstrated benign prostatic hyperplasia (Fig. 3a), and the pathology of the follicular lesions demonstrated chronic inflammation with denudation or loss of epithelium and marked inflammatory infiltration (Fig. 3b). During the one-year follow-up, the patient quit ketamine completely and did not report any complaints except mild frequency which did not trouble him much, and there were no voiding or sexual complaints. Additionally, hydronephrosis and hydroureter were alleviated.

## Discussion and conclusions

Prior to 2018, there were approximately 8 reported cases of male patients aged 30 or younger diagnosed with BPH in the literature, as listed in Table 1 [1–8].

**Table 1:** BPH in children or young men < 30 years old

author	time	age	symptoms	PSA	treatment	comments
Powell	1939	17	dysuria	not mentioned	conservative treatments	gonadotrophin administration for the treatment of cryptorchidism.
Sumiya	1987	20	Dysuria, pain in the perineal area	not mentioned	suprapubic prostatectomy, excisional tissues 380 g	
Harada	1989	28	Dysuria, haematuria	not mentioned	suprapubic prostatectomy, excisional tissues 31 g	
Romano	2001	23	Dysuria, acute urinary retention, urinary incontinence, enuresis	not mentioned	TURP, excisional tissues approximately 38 g	Combined with acromegaly, height 2.03 m.
Choi	2005	10	Dysuria, uroschesis, haematuria	0.02 ng/ml.	TURP, excisional tissues approximately 33 g	The same patient was diagnosed with BPH at 10 and 13 years old.
		13	Dysuria, fever, weakness	0.04 ng/ml	suprapubic prostatectomy, excisional tissues 55 g	
Georgiades	2014	27	LUTS, haemospermia	0.33 ng/ml	TURP, excisional tissues 181 g	with spermatocystitis, retrograde ejaculation after TURP.
Yagmur	2016	17	Dysuria, acute urinary retention, haematuria	not mentioned	TURP	prostatic hyperplasia of the middle lobe.
Zouari	2018	22	Dysuria, acute urinary retention	1.6 ng/ml	TURP, excisional tissues approximately 60 g	biopsy indicated glandular cystitis, but pathological examination after operation demonstrated BPH.

BPH: benign prostatic hyperplasia

PSA: prostate-specific antigen

TURP: transurethral resection of the prostate

LUTS: lower urinary tract symptoms

Of these cases, the youngest was 10 years old, while the oldest was 29 years old. In most cases, the cause was not described. However, some authors illuminated possible pathogenic factors as causes in their cases as follows: Powell [1] reported a man aged 17 years old who developed benign prostatic hyperplasia after gonadotrophin therapy due to cryptorchidism. A case of a 23-year-old man who was admitted with acute urinary retention indicating BPH was reported by Romano et al. [4], who investigated the possible relationship between BPH and acromegaly due to the 2.03-m height of the patient. Zouari et al. [8] reported a 22-year-old man with acute urinary retention caused by BPH whose biopsy during examination indicated glandular cystitis. Unexpectedly, the pathological examination after transurethral resection of the median lobe of the prostate showed BPH. The author concluded that further research was needed to determine the correlation between BPH and cystitis glandularis. Eight patients underwent surgery for open suprapubic transvesical prostatectomy or transurethral resection of the prostate (TURP). Only one patient underwent nonoperative treatment who ever accepted treatment with gonadotropic hormones due to cryptorchidism before the occurrence of BPH [1]. The patient in our case had no history of endocrinological disease or other possible relevant factors except 3-year ketamine inhalation.

Prostate specific antigen (PSA) seems to represent a prominent difference in BPH between elderly men and young males. According to the cases listed in Table 1, all young BPH patients were characterized by a normal PSA range except those who did not have reported PSA levels, and in our case, the PSA level was 1.80 g/ml. Elderly patients usually have a PSA level that is higher than the normal range.

To our knowledge, there has been no case of BPH with ketamine-associated uropathy ever reported in young patients. The exact pathophysiology of ketamine-associated cystitis remains greatly unknown. An important theory is that ketamine and/or its metabolites accumulates at a high concentration in urine, leading to a direct toxic effect on the urothelium and resulting in significant inflammation. The nuclear factor-kappa B (NF-κB) pathway was found to be activated during inflammatory signalling of ketamine-induced cystitis in the bladder in a rat model [9]. Furthermore, there exists a significant increase in pro-inflammatory cytokines, such as IL-1β, IL-2, IL-4, IL-6, IFN-γ, NGF, and COX-2, in bladder tissue in ketamine-associated cystitis [10, 11]. Similarly, although ageing and androgens are the two established risk factors in the progression of benign prostatic hyperplasia (BPH), the pathogenesis of BPH is still largely unresolved. Currently, increasing evidence indicates that inflammation is strongly involved in the aetiology and progression of BPH. Particularly, it has been identified that inflammatory cytokines, IL­2, IL­4, IL­7, IL­17, IFN-γ and their relevant receptors are upregulated in and that inflammatory cells infiltrate into BPH tissues [12–14]. Anti-inflammatory agent was proved to produce an antiproliferative effect during BPH by markedly decreased BPH-related upregulation of COX-2 protein expression [15]. It is possible that inflammation in the urinary tract due to the direct and indirect effects of ketamine stimulates the enlargement of the prostate. However, few studies have investigated the direct effect of ketamine or its metabolites on the prostate, and their relationship needs to be confirmed with further research.

Ketamine-induced upper urinary tract lesions seem not to be paid enough attention due to a greater incidence of apparent lower urinary tract syndromes [16]. Hydronephrosis and ureteral wall thickening were reported most frequently in ketamine-induced upper urinary tract damage. According to clinical case summaries, the incidence of hydronephrosis (bilateral or unilateral) can be as high as 44.4% [17] or 51% [18]. Huang LK et al. [17] reported three patients with unilateral hydronephrosis and nine with bilateral hydronephrosis among twelve ketamine-induced uropathy patients with hydronephrosis. In addition, nine patients had ureteral wall thickening, and two had ureterovesical junction involvement. Bilateral upper ureteric narrow and mild bilateral hydronephrosis were detected in 3 patients in a 6-patients cases report [19]. Misra S et al. [20] reported that two of 34 patients had bilateral hydronephrosis with hydroureters to the vesicoureteral junction. In another case of A 26-year-old man with ketamine abuse, bilateral hydronephrosis, obstructive nephropathy and kidney injury were detected in spite of no obstruction at the ureteric orifices [21]. Many other studies referred to bilateral or unilateral hydronephrosis in ketamine-related uropathy but did not report the ureteric or renal lesions [18, 22]. In our case, bilateral moderate hydronephrosis with hydroureters to the vesicoureteral junction was detected, but ureteral wall thickening or ureteric narrow was not found. Therefore, obstruction due to BPH and bladder damage in anatomy and function may be the primary causes of hydronephrosis and hydroureters in our case.

The general treatment of BPH in elderly men includes medical and surgical approaches. Medical approaches may not work due to the indefinite aetiology of BPH in young men. For ketamine-associated uropathy, the aims of management of ketamine-associated urinary symptoms are symptom control and preservation of renal function and are ultimately to prevent irreversible damage to the urinary tract [23]. Due to the small number of cases of BPH in young people, there has been no consensus established on treatment. However, as shown in Table 1, most cases of children or young adults with BPH underwent surgery therapy, and surgical intervention may be necessary in most cases. Meanwhile, avoiding potential or possible pathogenic factors (ketamine in our case) are also necessary during the following treatment.

The simultaneous occurrence of BPH and ketamine-associated uropathy in young males has never been reported before. Inflammation in the urinary system, as a consequence of ketamine, may contribute to the development of BPH. Both surgical interventions and ketamine cessation are important approaches for relieving symptoms. This case may deserve significant attention as ketamine addiction among young population increases globally.

## Data Availability

The supporting data and materials can be found in the file cabinet in the department of urology of Xiangya Hospital Central South University. These confidential patient data within the patient’s information should not be shared to the public. The datasets used in this case report are available from the corresponding author on reasonable request.

## Abbreviations

BPH: Benign prostate hyperplasia
CT: Computed tomography
PSA: Prostate specific antigen
TURP: Transurethral resection of the prostate
